# Preoperative mediastinal staging in patients with cT1‐3NxM0 non‐small cell lung cancer

**DOI:** 10.1111/1759-7714.13673

**Published:** 2020-10-07

**Authors:** Bastiaan E. Steunenberg, Tom P.A. Beddows, Hans G.W. De Groot, Ninos Ayez, Cor Van Der Leest, Joachim G.J.V. Aerts, Eelco J. Veen

**Affiliations:** ^1^ Department of Pulmonary Surgery Amphia Hospital Breda Breda The Netherlands; ^2^ Department of Pulmonary Medicine Amphia Hospital Breda Breda The Netherlands

**Keywords:** Mediastinum, NSCLC, preoperative staging, quality management

## Abstract

**Background:**

Endosonography is accepted as the initial procedure for mediastinal staging in patients with suspected non‐small cell lung cancer (NSCLC). However, the diagnostic value of different staging methods in specific subgroups is unclear. The purpose of this study was to assess the performance and outcome of mediastinal staging in lung cancer in a general teaching hospital.

**Methods:**

The records of 870 consecutive patients with potentially resectable NSCLC (cT1‐3NxM0) were analyzed in a retrospective cohort study between January 2010 and December 2016. Patients were divided into four different groups according to ESTS guidelines. The primary endpoint was the rate of unforeseen mediastinal metastasis in these groups and the sensitivity of different staging methods.

**Results:**

Mediastinal staging was performed in 336 patients of whom 112 (33%) underwent lobectomy. Unforeseen mediastinal metastasis was seen in 10 (9%) patients after negative mediastinal staging. Sensitivity after combined mediastinal staging (endosonography with mediastinoscopy) in the overall group was 94%. In patients without suspected mediastinal lymph nodes but with suspected hilar lymph nodes (N1), or a peripheral tumor >3 cm, sensitivity of endosonography was 33% and mediastinoscopy 75%. Biopsy of at least level 4L, 4R and 7 was taken in 18% of the endosonographies and 58% of the mediastinoscopies.

**Discussion:**

Combined mediastinal staging (endosonography with mediastinoscopy) is reliable with a sensitivity of 94%. However, the diagnostic value of endosonography in patients with suspected hilar lymph nodes or a peripheral tumor >3 cm is questionable, and in these patients, performing direct mediastinoscopy should be considered.

**Key points:**

**Significant findings of this study:**

The diagnostic value of endosonography in patients without suspected mediastinal lymph nodes but with potential risk factors (suspected N1 disease or peripheral tumor >3 cm) is questionable. Therefore, mediastinoscopy as the first choice should be considered in these patients.

**What this study adds?:**

Accurate mediastinal nodal staging is essential in patients with suspected NSCLC to avoid unnecessary lobectomy. Detailed knowledge about sensitivity and specificity of mediastinal staging techniques in different patient groups can make a difference.

## Graphical abstract

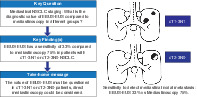

EBUS‐EUS has a sensitivity of 33% compared to mediastinoscopy, 75% in patients with cT1‐3N1 or cT2‐3N0 NSCLC. The value of EBUS‐EUS must be questioned in cT1‐3N1 or cT2‐3N0 patients, and direct mediastinoscopy should be considered.

## Introduction

Non‐small cell lung cancer (NSCLC) is the most frequently diagnosed cancer worldwide.^1^ Mediastinal lymph node status is an important determinant in the prognosis and choice of therapy.^2^, ^3^ If N2 disease has been proven in patients with positive mediastinal lymph nodes, generally they are no longer eligible for surgical therapy.

For decades, mediastinoscopy has traditionally been the gold standard for mediastinal staging with sensitivity between 75% and 95%.^4^, ^5^ Over the past decades, less invasive techniques, such as endobronchial and esophageal endosonography have been introduced for mediastinal staging. At first, endosonography was only used as a tissue sampling procedure but nowadays it is used as a tool for systematic mediastinal staging. In 2015, the European Society of Thoracic Surgery (ESTS) recommended endosonography as the initial procedure for mediastinal nodal staging in patients with suspected or proven NSCLC.^6^ The preferred endosonographic investigation is a combination of endobronchial ultrasound with real‐time guided transbronchial needle aspiration (EBUS‐TBNA) and endoscopic esophageal ultrasound with fine needle aspiration (EUS‐FNA). These two techniques combined can result in a sensitivity of 85% in patients with enlarged or fluorodeoxyglucose PET‐CT (FDG PET‐CT)‐avid mediastinal lymph nodes if well performed. Adding mediastinoscopy has been reported to increase sensitivity up to 94%.^4^ Patients with suspected hilar lymph nodes or a tumor of >3 cm have a potentially higher risk of mediastinal metastasis of 6%–30%.^6^ Therefore, accurate mediastinal nodal staging in these patients is warranted.

The outcome of mediastinal lymph node staging is dependent on the interpretation of the FDG PET‐CT scan and thoroughness and performance of the procedure according to current guidelines.^7^ Sampling from at least three lymph node stations is recommended for both mediastinoscopy and endosonography.^6^, ^8^

The aim of this study was to assess the performance and outcome in daily practice of the mediastinal staging process in patients with suspected cT1‐3NxM0 NSCLC in a general teaching hospital according to current ESTS guidelines.

## Methods

In this retrospective cohort study, the medical records of 870 consecutive patients with potentially resectable NSCLC were included (T1‐3NxM0), according to the International Union Against Cancer Tumor Node Metastasis classification version 7. Patients with proven distant metastases (M1) or locally advanced T4 NSCLC were excluded. All patients were diagnosed and had their primary work‐up for suspected NSCLC in the Amphia Hospital located in Breda, the Netherlands, between January 2010 and December 2016. Population based data from the southern region of the Netherlands Cancer Registry (NCR by IKNL) were used. The NCR received notifications of all newly diagnosed malignancies by the automated pathology archive (PALGA).

Standard work‐up for surgically fit patients was performed by a pulmonologist according to Dutch guidelines and included the following investigations: integrated FDG PET‐CT scan, bronchoscopy and lung function tests. If bronchoscopy revealed no abnormality, an additional transthoracic biopsy of the lesion was performed when technically feasible. A multidisciplinary team consisting of a pulmonologist, thoracic surgeon, radiologist, radiotherapist and nuclear medicine physician discussed all patients.

Patients were divided into four different clinical staging groups according to the ESTS guidelines^6^ and FDG PET‐CT imaging: Group 1: Patients with suspected mediastinal lymph nodes (regardless of the tumor location). Group 2: Patients without suspected mediastinal lymph nodes but on the FDG PET‐CT there were (i) suspected ipsilateral hilar lymph nodes; or (ii) peripheral tumor > 3 cm. Group 3: Patients with a central tumor without hilar or mediastinal lymph nodes. Group 4: Patients with a peripheral tumor < 3 cm and without hilar or mediastinal lymph nodes.

### Mediastinal staging

According to the ESTS guidelines, additional mediastinal staging was performed in cases of enlarged (>10 mm short axis) and/or FDG PET‐CT positive mediastinal lymph nodes. In patients without mediastinal involvement on FDG PET‐CT, additional mediastinal staging was performed in patients with suspected ipsilateral hilar lymph nodes, a centrally located tumor and tumors >3 cm.

The first step in further mediastinal investigation was by endoscopic ultrasonography (EBUS‐TBNA or/and EUS‐FNA) which was performed under conscious sedation by trained pulmonologists. Reachable lymph nodes were assessed during the procedure and suspected lymph nodes were biopsied. In order to predict malignancy, lymph nodes were assessed by different characteristics. These characteristics were: shape (round vs. oval); size (>10 mm vs. <10 mm longest short axis); and echogenicity (hypoechoic vs. hyperechoic appearance). Patients were further scheduled for cervical mediastinoscopy in cases of a negative or nonrepresentative result of the biopsied lymph nodes after endosonography. Cervical mediastinoscopy was performed using video‐assisted mediastinoscopy (VAM; Karl Storz) in all patients. A systematic examination of the mediastinum was performed following the ESTS guidelines.^6^ Anatomical lung resection was performed by video‐assisted thoracoscopic surgery (VATS) or thoracotomy with lymph node sampling or lobe‐specific lymph node dissection after negative mediastinoscopy.

All surgical and pathology reports were analyzed with regard to the assessed or biopsied lymph node stations following endosonography (EBUS, EUS or EBUS‐EUS), mediastinoscopy and surgical resection.

The end point of this study was the rate of unforeseen presence of mediastinal nodal metastases after negative mediastinal staging and sensitivity of the different mediastinal staging techniques in the four different groups.

Statistical analysis was performed using SPSS 20 statistical software. Descriptive statistics were used to describe patient characteristics and outcome. Sensitivity and negative predictive value (NPV) were calculated using the standard formulas.

## Results

### Patient characteristics

**Figure 1:**
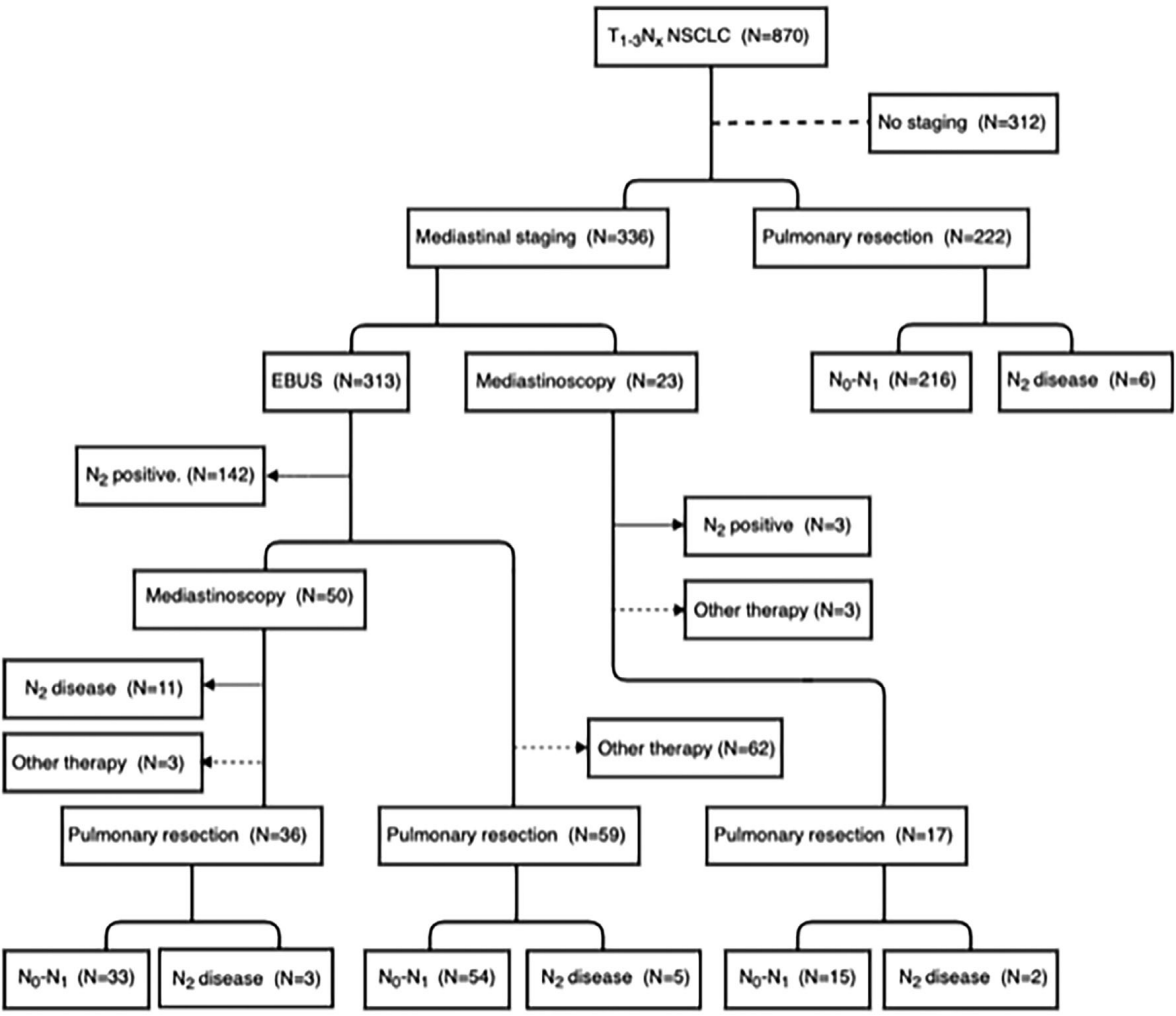
Flow diagram of all 870 patients with potentially resectable T1‐3NxM0 NSCLC. Mediastinal staging was performed in 336 patients (left side of the diagram) and upfront surgery without mediastinal staging was performed in 222 patients (right side of the diagram).

We excluded 312 (36%) of the 870 patients because they were unfit for surgery and mediastinal staging was not performed (Table 1). These patients opted for chemotherapy/radiotherapy or no further treatment. Upfront surgery without preoperative mediastinal staging was performed in 222 patients (25%) (Table 1, Fig 1).

**Table 1:** Patient characteristics of 558 patients with suspected T1‐3NxM0 non‐small cell lung cancer (NSCLC) after diagnostic work‐up for surgery

Male/female		*N* = 500 (57%) / *N* = 370 (43%)
Age (mean)		69 (40–81)
	**Mediastinal staging (*N* = 336)**	**Direct pulmonary resection (*N* = 222)**
ESTS guideline groups:		
Group 1 (*N* = 220)	215 (98%)	5 (2%)
Group 2 (*N* = 166)	78 (47%)	88 (53%)
Group 3 (*N* = 29)	24 (83%)	5 (17%)
Group 4 (*N* = 143)	19 (13%)	124 (87%)
Clinical stage:		
Stage IA/B (*N* = 227)	46 (20%)	181 (80%)
Stage IIA/B (*N* = 71)	48 (68%)	23 (32%)
Stage IIIA (*N* = 148)	145 (98%)	3 (2%)
Stage IIIB (*N* = 51)	50 (98%)	1 (2%)
Unknown (*N* = 61)	47 (77%)	14 (23%)

Mediastinal staging by EBUS, EUS, mediastinoscopy or a combination of these techniques was performed in 336 patients (39%). Mediastinal staging in group 1 (patients with suspected mediastinal lymph nodes) was performed in 215 patients (64%), in group 2 (patients without suspected lymph nodes but with suspected N1 nodes or peripheral tumor >3 cm) in 78 patients (23%) and in group 3 (patients with a central tumor without suspected mediastinal lymph nodes) in 24 patients (7%). After negative mediastinal staging, a lobectomy was performed in 112 patients (33%).

### Outcome and performance of mediastinal staging in the overall group (*N* = 336)

Endosonography was the first choice for mediastinal staging in 313 patients (93%). For unknown reasons, mediastinoscopy was the first choice to for mediastinal staging in 23 (7%) patients. The latter group revealed three patients (13%) with N2 disease.

In those patients who underwent an endosonography, N2 disease was detected in 142 (45%). In the patients who had a negative endosonography (*N* = 171), a mediastinoscopy was performed in 50 patients (29%) and pulmonary resection in 59 patients (35%). The remaining 62 patients (36%), with a negative endosonography, were unfit for surgical therapy and opted for radiotherapy, or no further treatment.

With endosonography, a complete assessment of the mediastinum with verification or sampling of at least stations 4R, 4L and 7 and the suspected lymph node station was performed in 57 patients (18%). Endosonography was not representative in 10% (*N* = 31).

Mediastinoscopy was performed in 73 patients (23 patients as first choice and 50 patients after endosonography). With mediastinoscopy, a complete assessment of the mediastinum with sampling of at least stations 4R, 4L and 7 and the suspected lymph node station was done in 42 patients (58%). In the 50 patients where mediastinoscopy had been performed, following negative endosonography, N2 disease was revealed in 11 patients (22%): six patients with suspected lymph nodes (group 1) and five patients without suspected lymph nodes, but with a risk factor (group 2). These positive lymph nodes were biopsied during endosonography in seven patients but had negative (three patients), or no representative results (four patients). In the other four patients, no tissue samples were taken with endosonography. In these 11 patients, a complete mediastinal assessment with endosonography was carried out in only two patients.

After a negative mediastinoscopy, 53 patients underwent pulmonary resection and six patients did not undergo surgery. These six patients opted for radiotherapy or chemotherapy after consultation due to comorbidities.

### Outcome of anatomical surgical resection

Anatomical surgical resection was eventually performed in 334 patients. Lymph node sampling was performed in 167 patients (50%) and lobe‐specific lymph node dissection in 165 patients (50%) (Table 2). Up front surgery without preoperative mediastinal staging was performed in 222 patients, revealing unforeseen N2 disease in six patients (2.7%). Surgical resection after negative mediastinal staging was performed in 112 patients, revealing unforeseen N2 disease in 10 patients (8.9%).

**Table 2:** Surgical and histopathological outcome after anatomical resection

Choice of therapy during lobectomy	
Lobe‐specific lymph node dissection	*N* = 167
Lymph node sampling	*N* = 165
Final histopathology after lobectomy	
Adenocarcinoma	*N* = 187
Squamous cell carcinoma	*N* = 127
Large cell carcinoma	*N* = 9
Carcinoma not further specified	*N* = 11

### Post‐procedural N‐status

In all patients, the sensitivity in detecting mediastinal nodal metastases after total mediastinal staging was 94% (NPV 91.1%), for endosonography alone 88.2% (NPV 80.0%), and mediastinoscopy alone 73.7% (NPV 90.6%) in the overall group (Table 3). In group 1 (*N* = 210) patients with suspected mediastinal lymph nodes, sensitivity of mediastinal staging was 99% (NPV 97%) with sensitivity of endosonography 94.9% (NPV 82.5%) and mediastinoscopy 100% (NPV 100%), respectively. In group 2 (*N* = 78) (patients without suspected mediastinal lymph nodes but with suspected N1 lymph nodes or peripheral tumor >3 cm) sensitivity of mediastinal staging was 71% (NPV 92%) with sensitivity of endosonography 33% (NPV 83.7%) and mediastinoscopy 75% (NPV 87.5%), respectively. In group 3 (*N* = 24) (patients with a central tumor without suspected mediastinal lymph nodes) sensitivity of mediastinal staging was 75% (NPV 75%) with sensitivity of endosonography 80% (NPV 80%) and mediastinoscopy 25% (NPV 62.5%), respectively.

**Table 3:** Diagnostic performance and unforeseen N2 disease in patients after mediastinal staging divided into different groups

	Overall	Group 1 (*N* = 210)	Group 2 (*N* = 78)	Group 3 (*N* = 24)
Diagnostic performance:				
Total mediastinal staging:				
Sensitivity	94%	99%	71%	75%
Negative predictive value	91%	97%	92%	75%
Endosonography:				
Sensitivity	88%	95%	33%	80%
Negative predictive value	80%	83%	84%	80%
Mediastinoscopy:				
Sensitivity	74%	100%	74%	25%
Negative predictive value	91%	100%	88%	63%
Unforeseen N2 disease:				
During mediastinoscopy after negative endosonography (*N* = 50)	22%	20.1%	35.7%	0%
During surgery after negative staging (*N* = 112)	8.9%			
During direct surgery (*N* = 222)	2.7%	2.5%	7.8%	25%

## Discussion

In our department, the established guidelines of the ESTS^6^ are used in patients with cT1‐3NxM0 lung cancer. This study demonstrates that daily practice sensitivity was 88.2% for endosonography and 73.7% for mediastinoscopy. Combining these two techniques resulted in a sensitivity of 94%, which is comparable to the results of the ASTER‐trial.^4^ This study showed no significant difference between endosonography alone (sensitivity 85%) in comparison with direct surgical staging (sensitivity 79%), and the sensitivity of endosonography in combination with surgical mediastinal staging was 94%.^4^

However, in this period, only 18% of the endosonographies and 58% of the mediastinoscopies were performed according to the guidelines. Nowdays, we have implemented a structural approach and description of the nodes when performing endosonography as is already the case with mediastinoscopy.^8^

Endosonography is accepted as the first choice for mediastinal staging in patients with suspected mediastinal lymph nodes as seen on CT or FDG PET‐CT. The probability of having mediastinal metastasis in these patients has previously been reported to be 50%–80%.^5^ Gu *et al*. reported a sensitivity for EBUS alone of 94% in patients with an abnormal mediastinum on imaging.^9^ Our study confirmed these results with sensitivity of 95% for endosonography alone in patients with PET‐CT suspected mediastinal lymph nodes.

The risk of mediastinal metastases in patients without suspected lymph nodes at PET‐CT but with enlarged or FDG‐avid hilar lymph nodes or lung tumor >3 cm has been reported to be 6%–30%.^6^ In a prospective multicentre study, Dooms *et al*. revealed that endosonography alone had a sensitivity of 38% in patients with cN1 disease on imaging and could be increased to 73% by adding mediastinoscopy.^10^, ^11^ Our study showed similar results as endosonography alone had a sensitivity of 33% in patients with suspected N1 disease or lung tumor >3 cm and sensitivity increased to 71% when followed by mediastinoscopy. Therefore, the yield of endosonography in this group was questionable and direct mediastinoscopy for mediastinal staging might be more appropriate. In our institute, the guideline was followed in only 47% (Table 1) of the cases in patients without suspected mediastinal lymph nodes but with potential risk factors.

The ESTS guidelines recommend exploration of the mediastinum in centrally located lung tumors (without suspected mediastinal or hilar lymph nodes) as the false negative rates of CT and FDG PET‐CT imaging are high (20%–25% and 24%–83%, respectively).^6^ Endosonography is the preferred staging technique for the mediastinum based on expert opinion. In our study, the sensitivity of mediastinal staging was 75% in patients with centrally located lung tumors with a sensitivity of endosonography and mediastinoscopy of 80% and 25%, respectively. However, these results might not be representative due to the very small number of patients.

This study found that in our hospital only 18% of endosonographies and 58% of mediastinoscopies were executed according to the ESTS guidelines which included assessment of at least stations 4L, 4R and 7. These results are in line with other studies which demonstrated that only 40%–50% of the cases underwent a mediastinoscopy according to the guidelines.^8^, ^12^ To date, endosonography data is scarce. These results could be explained by the fact that the physician performing the procedure only focused on suspected lymph nodes, instead of taking biopsies of the mediastinum in a systematic approach.

In our study, 50 patients underwent subsequent mediastinoscopy after negative endosonography identifying another 11 patients with N2 disease. In seven of those patients, sampling of the positive lymph node at mediastinoscopy was performed during endosonography but results were not representative or (false) negative. This was similar to the results of Verhagen *et al*.^13^ indicating a sampling error during endosonography.

One of the strengths of this study was the large size of the cohort group. Moreover, this cohort provided representative information of daily practice in a general hospital. A limitation of this study was that in some patients during lobectomy only lymph node sampling was performed instead of systematic lymph node dissection. Theoretically, this might have underestimated N2 disease in patients after negative mediastinal staging. Another limitation was the retrospective nature of this study. However, we do believe that the data presented here provides true information of daily practice and incites us to improve the quality of invasive mediastinal staging techniques.

Our study demonstrated that there is a reliable role for endosonography in mediastinal staging in patients with cT1‐3NxM0 suspected NSCLC. Although endosonography and mediastinoscopy were not always performed according to the current guidelines, sensitivity was comparable to that previously reported in the literature. A critical concern was the diagnostic value of endosonography in patients without suspected mediastinal lymph nodes but with potential risk factors (suspected N1 disease or peripheral tumor >3 cm) as our study showed a sensitivity of only 33% in this group of patients. Therefore, mediastinoscopy should be considered as the first choice in these patients.

The outcome of different studies are difficult to compare, because not all clinics adhere to the same guidelines. By implementing systematic endosonography in the near future, we hope to change current practice. This will make it easier to compare the results between hospitals and to use these in future research.

## Disclosure

There are no potential conflicts of interest, including specific financial interests and relationships and affiliations.
